# Supplementary material for: Detection of hepatitis viruses in suspected cases of Viral Haemorrhagic Fevers in Nigeria
Source: PLoS One. 2024 Jun 21;19(6):e0305521. doi: 10.1371/journal.pone.0305521 (PMC11192311; doi:10.1371/journal.pone.0305521)
Supplement: S1 Raw images — (PDF) [file pone.0305521.s001.pdf]

HBV (S-GENE)

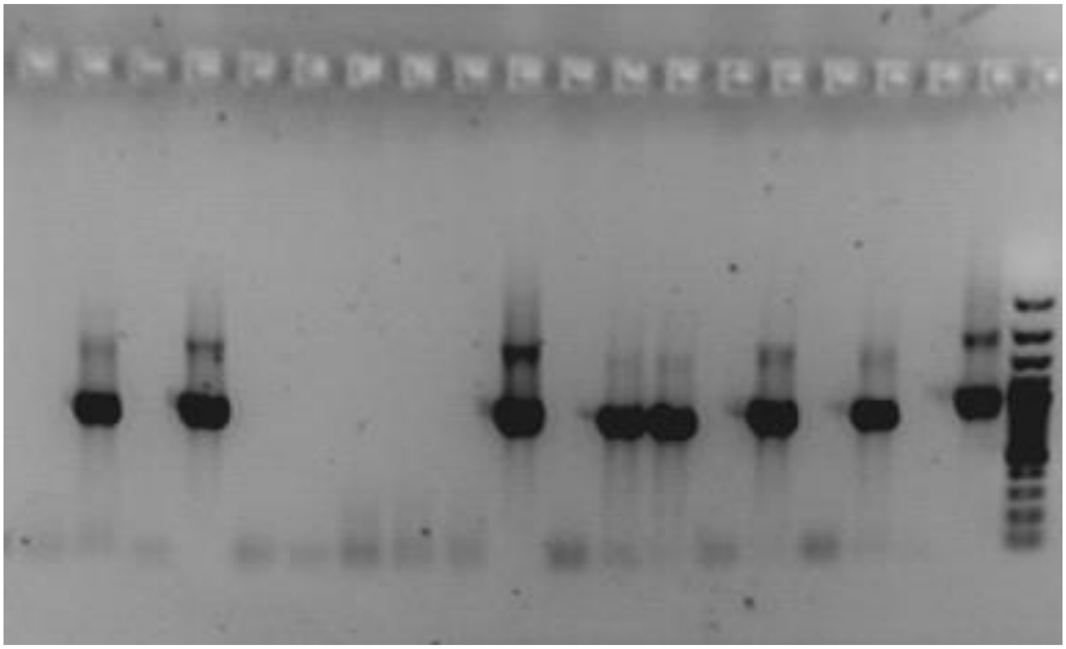

1 2 3 4 5 6 7 8 9 10 11 12 13 14 15 16 17 N P L

N = NEGATIVE CONTROL

P = POSITIVE CONTROL

L = 100bp LADDER

Samples number 2,4,10,12,13,15 and 17 are positives with amplicon size of 1000+bp

P2f (5′-CCTGCTGGTGGCTCCAGTTC-3′*)*

Mc2r (rv) TGGAAGTTGGGGATCATTGC

HEV

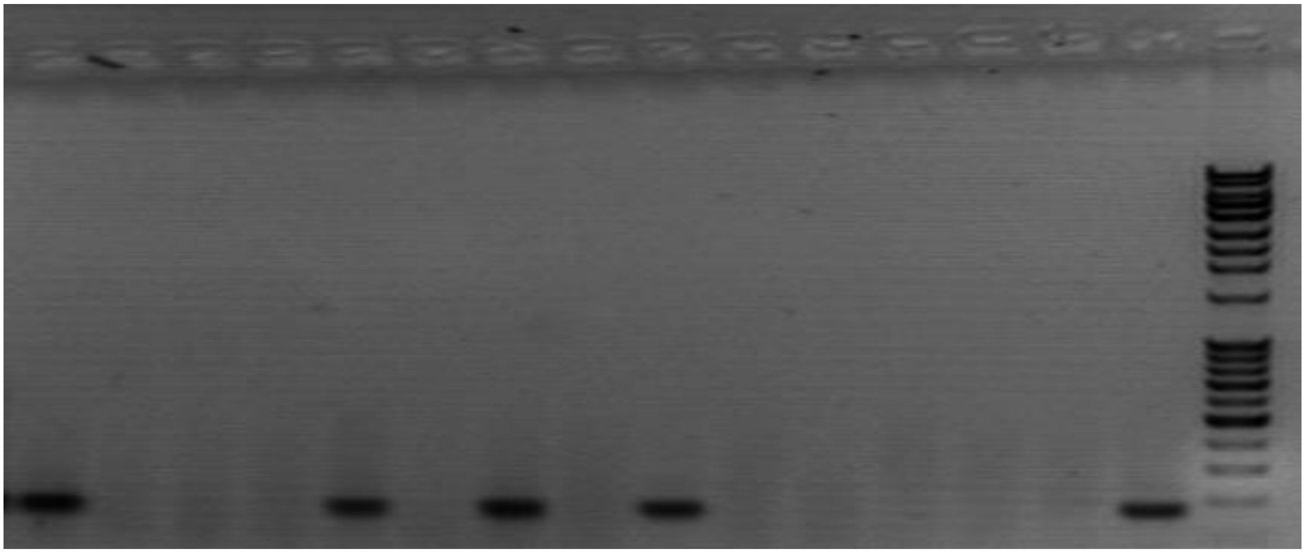

1 2 3 4 5 6 7 8 9 10 11 12 13 N P L

N = NEGATIVE CONTROL

P = POSITIVE CONTROL

L = 100bp LADDER

Sample no 1,5,7 and 9 are positives with amplicon size of 170bp

ORF1 FN Forward nested CTCCTGGCRTYACWACTGC

ORF1 RN Reverse nested GGRTGRTTCCAIARVACYTC

HCV

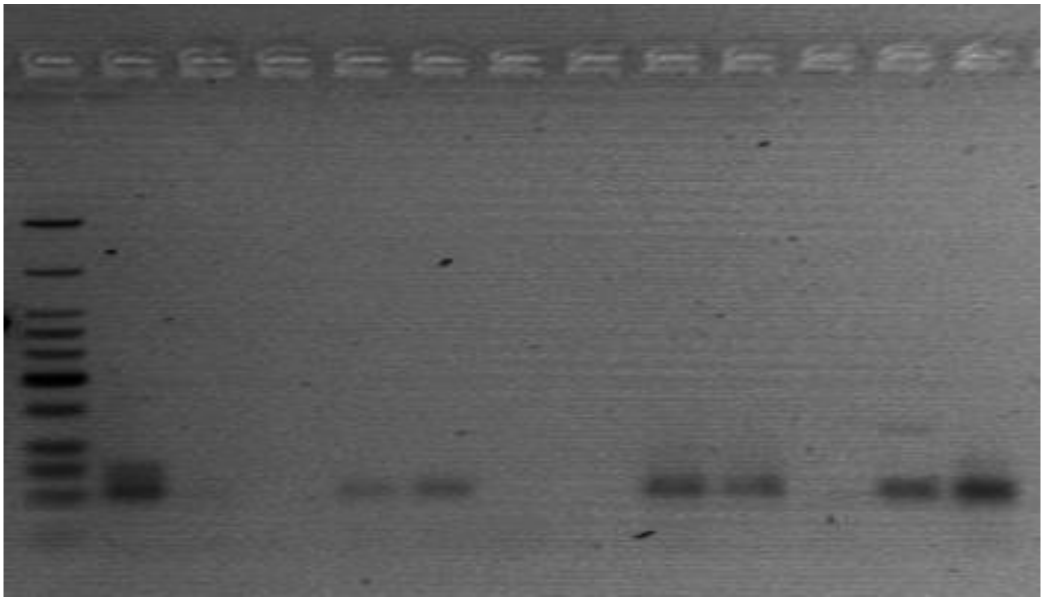

L 1 2 3 4 5 6 7 8 9 N P1 P2

N = NEGATIVE CONTROL

P = POSITIVE CONTROL

L = 100bp LADDER

Sample no 1, 4,5,8 and 9 are positives with amplicon size of 200bp

HCV A2 GCGACCCAACACTACTCGGCT

HCV S2 ATGGCGTTAGTATGAGTG

HAV

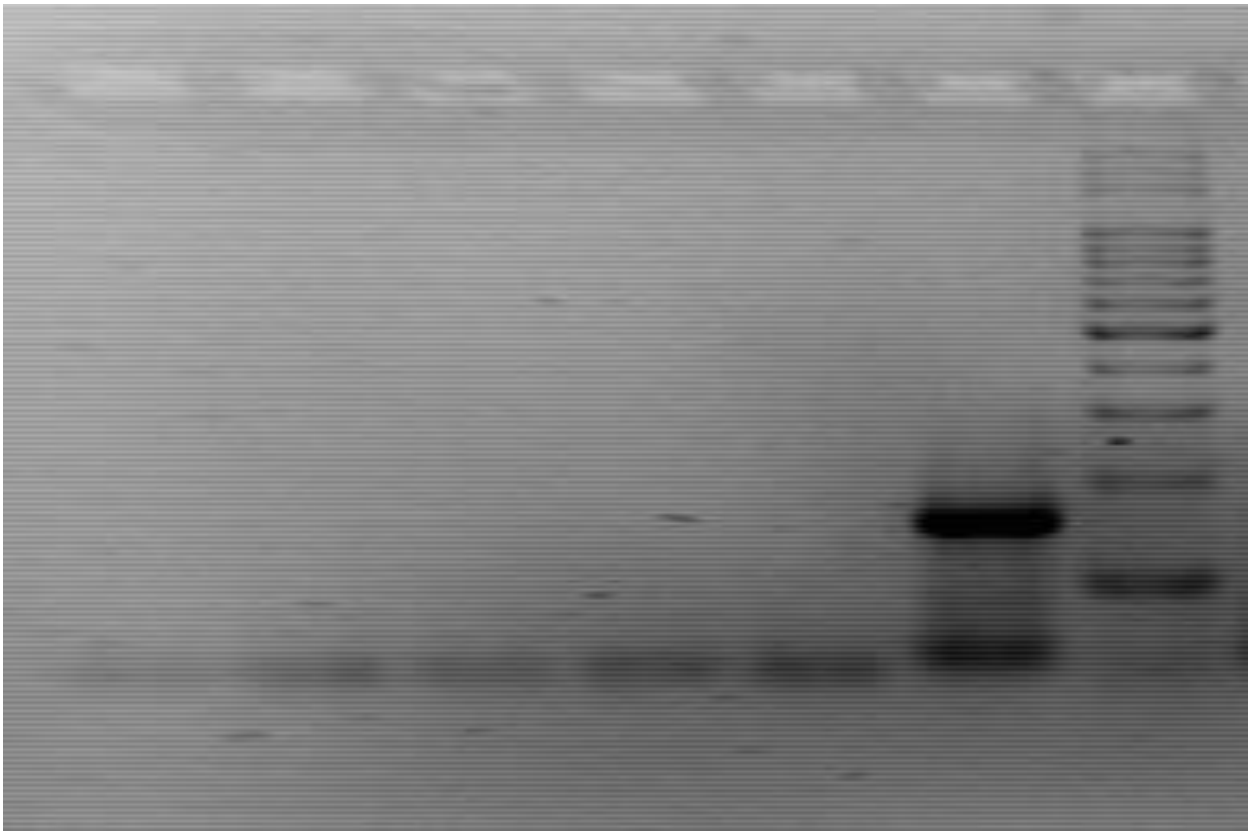

1 2 3 4 N P L

N = NEGATIVE CONTROL

P = POSITIVE CONTROL

L = 100bp LADDER

They are all negative with Amplicon size of 192bp

HAVC-R, 5'-CTCCAGAATCATCTCCAAC-3'

HAVC-L, 5'-CAGCACATCAGAAAGGTGAG-3'
