# Supplementary material for: Detection of hepatitis viruses in suspected cases of Viral Haemorrhagic Fevers in Nigeria
Source: PLoS One. 2024 Jun 21;19(6):e0305521. doi: 10.1371/journal.pone.0305521 (PMC11192311; doi:10.1371/journal.pone.0305521)
Supplement: Attachment — Submitted filename: PONE-D-23-34199.pdf [file pone.0305521.s002.pdf]

PLOS ONE

Detection of Hepatitis Viruses in Suspected Cases of Viral Haemorrhagic Fevers in Nigeria

--Manuscript Draft--

**Manuscript Number:**	PONE-D-23-34199
**Article Type:**	Research Article
**Full Title:**	Detection of Hepatitis Viruses in Suspected Cases of Viral Haemorrhagic Fevers in Nigeria
**Short Title:**	Hepatitis Viruses in Suspected Cases of Viral Haemorrhagic Fevers in Nigeria
**Corresponding Author:**	Olumuyiwa Babalola Salu, Ph.D.<br>University of Lagos College of Medicine<br>Idi-Araba, Lagos NIGERIA
**Keywords:**	Hepatitis A Virus, Hepatitis B Virus, Hepatitis C Virus, Hepatitis E Virus, Viral Haemorrhagic fever, Enzyme Immunoassay, Polymerase Chain Reaction.
**Abstract:**	There have been several Viral Hemorrhagic Fever (VHF) outbreaks in Nigeria which remains a public health concern. Despite the increasing number of suspected cases of VHF due to heightened surveillance activities and growing awareness, only a few cases are laboratory-confirmed to be VHF. Routinely, these samples are only tested for Lassa virus and Yellow fever virus with occasional testing for Dengue virus when indicated. The aetiology of the disease in these VHF suspected cases in Nigeria which are negative for Lassa, Yellow fever and Dengue viruses remains a puzzle. Since the clinical features exhibited by suspected VHF cases are like other endemic illnesses such as Hepatitis, there is a need to investigate the diversity and co-infections of hepatitis viruses as differentials and possible co-morbidity in suspected cases of VHFs in Nigeria. A total of three hundred and fifty (350) blood samples of 212 (60.6%) males and 138 (39.4%) females ages ranged <1 - 70 years and mean age of 25 ±14.5 suspected of VHFs were analyzed for Hepatitis A, B, C and E viruses at the Centre for Human and Zoonotic Virology (CHAZVY), College of Medicine, University of Lagos (CMUL) using serologic and molecular techniques. The overall Enzyme Immunoassay Assay analysis revealed that 126 (36%) were positive for hepatitis. Individual prevalence revealed that none (0%) was reactive for HAV, while 37 (10.6%), 18 (5.1%) and 71 (20.3%) were positive for HBV, HCV and HEV respectively. A co-infection rate of 11.9% was also observed, with HCV/HEV co-infections being the most prevalent and the Northern region having the greatest burden of infection. The evidence of hepatitis virus infections in suspected cases of VHF was documented. Thus, their associations as co-morbidities and/or mortalities in this category of individuals require further investigations in endemic countries such as Nigeria. Therefore, the possible inclusion of screening for hepatitis viruses and other aetiologic agents that could mimic infections in suspected cases of VHFs in Nigeria should be thoroughly evaluated to guide informed policy on the diagnosis and management of these cases.
**Order of Authors:**	Olumuyiwa Babalola Salu, Ph.D.<br>Tomilola Feyikemi Akinbamiro, MSc<br>Remilekun Mercy Orenolu, MSc<br>Onyinye Dorothy Ishaya, MSc<br>Roosevelt Amaobichukwu Anyanwu, MSc<br>Olubunmi Rita Vitowanu, MSc<br>Maryam Abiodun Abdullah, MSc<br>Adenike Helen Olowoyeye, MSc<br>Sodiq Olawale Tijani, MSc<br>Kolawole Solomon Oyedeji, Ph.D<br>Sunday Aremu omilabu, Ph.D

Powered by Editorial Manager® and ProduXion Manager® from Aries Systems Corporation

Additional Information:	
**Question**	**Response**
**Financial Disclosure**<br><br>Enter a financial disclosure statement that describes the sources of funding for the work included in this submission. Review the submission guidelines for detailed requirements. View published research articles from *PLOS ONE* for specific examples.<br><br>This statement is required for submission and **will appear in the published article** if the submission is accepted. Please make sure it is accurate.<br><br>**Funded studies**<br>Enter a statement with the following details:<br>• Initials of the authors who received each award<br>• Grant numbers awarded to each author<br>• The full name of each funder<br>• URL of each funder website<br>• Did the sponsors or funders play any role in the study design, data collection and analysis, decision to publish, or preparation of the manuscript?<br><br>Did you receive funding for this work?	The author(s) received no specific funding for this work.
**Competing Interests**<br><br>Use the instructions below to enter a competing interest statement for this submission. On behalf of all authors, disclose any competing interests that could be perceived to bias this work—acknowledging all financial support and any other relevant financial or non-financial competing interests.<br><br>This statement is required for submission and **will appear in the published article** if the submission is accepted. Please make sure it is accurate and that any funding sources listed in your Funding Information	The authors have declared that no competing interests exist.

later in the submission form are also declared in your Financial Disclosure statement.<br><br>View published research articles from *PLOS ONE* for specific examples.<br><br>**NO authors have competing interests**<br><br>Enter: *The authors have declared that no competing interests exist.*<br><br>**Authors with competing interests**<br><br>Enter competing interest details beginning with this statement:<br><br>*I have read the journal's policy and the authors of this manuscript have the following competing interests: [insert competing interests here]*<br><br>* typeset	
**Ethics Statement**<br><br>Enter an ethics statement for this submission. This statement is required if the study involved:<br><br>• Human participants<br>• Human specimens or tissue<br>• Vertebrate animals or cephalopods<br>• Vertebrate embryos or tissues<br>• Field research<br><br>Write "N/A" if the submission does not require an ethics statement.<br><br>General guidance is provided below. Consult the submission guidelines for detailed instructions. **Make sure that all information entered here is included in the Methods section of the manuscript.**	The protocol for this study was reviewed and approved by the Health Research Ethics Committee (HREC) of the College of Medicine of the University of Lagos (CMUL) (Approval Number: CMUL/HREC/02/20/718) with exemption from informed consent as the data was collected, stored, and analyzed as part of outbreak response activity by the Nigerian Centre for Disease Control (NCDC) network of laboratories at the Centre for Human and Zoonotic Virology (CHAZVY) from which permission to use was obtained. All data were fully anonymized before the authors access, kept confidential and stored in password-protected computers. Personal identifiers were not extracted, and the dataset was anonymized before sharing and analysis.

Powered by Editorial Manager® and ProduXion Manager® from Aries Systems Corporation

**Format for specific study types**<br><br>**Human Subject Research (involving human participants and/or tissue)**<br>• Give the name of the institutional review board or ethics committee that approved the study<br>• Include the approval number and/or a statement indicating approval of this research<br>• Indicate the form of consent obtained (written/oral) or the reason that consent was not obtained (e.g. the data were analyzed anonymously)<br><br>**Animal Research (involving vertebrate animals, embryos or tissues)**<br>• Provide the name of the Institutional Animal Care and Use Committee (IACUC) or other relevant ethics board that reviewed the study protocol, and indicate whether they approved this research or granted a formal waiver of ethical approval<br>• Include an approval number if one was obtained<br>• If the study involved *non-human primates*, add *additional details* about animal welfare and steps taken to ameliorate suffering<br>• If anesthesia, euthanasia, or any kind of animal sacrifice is part of the study, include briefly which substances and/or methods were applied<br><br>**Field Research**<br><br>Include the following details if this study involves the collection of plant, animal, or other materials from a natural setting:<br>• Field permit number<br>• Name of the institution or relevant body that granted permission	
**Data Availability**<br><br>Authors are required to make all data underlying the findings described fully available, without restriction, and from the time of publication. PLOS allows rare exceptions to address legal and ethical concerns. See the PLOS Data Policy and FAQ for detailed information.	Yes - all data are fully available without restriction

A Data Availability Statement describing where the data can be found is required at submission. Your answers to this question constitute the Data Availability Statement and **will be published in the article**, if accepted.<br><br>**Important:** Stating 'data available on request from the author' is not sufficient. If your data are only available upon request, select 'No' for the first question and explain your exceptional situation in the text box.<br><br>Do the authors confirm that all data underlying the findings described in their manuscript are fully available without restriction?	
**Describe where the data may be found in full sentences. If you are copying our sample text, replace any instances of XXX with the appropriate details.**<br><br>• If the data are **held or will be held in a public repository**, include URLs, accession numbers or DOIs. If this information will only be available after acceptance, indicate this by ticking the box below. For example: *All XXX files are available from the XXX database (accession number(s) XXX, XXX.*).<br>• If the data are all contained **within the manuscript and/or Supporting Information files**, enter the following: *All relevant data are within the manuscript and its Supporting Information files.*<br>• If neither of these applies but you are able to provide **details of access elsewhere**, with or without limitations, please do so. For example:<br><br>*Data cannot be shared publicly because of [XXX]. Data are available from the XXX Institutional Data Access / Ethics Committee (contact via XXX) for researchers who meet the criteria for access to confidential data.*<br><br>*The data underlying the results presented in the study are available from (include the name of the third party*	All relevant data are within the manuscript and its Supporting Information files.

and contact information or URL).<br>• This text is appropriate if the data are owned by a third party and authors do not have permission to share the data.<br><br>* typeset	
Additional data availability information:	

Manuscript

Click here to access/download;Manuscript;December 2023_Updated_PLOS ONE Manuscript.docx

Detection of Hepatitis Viruses in Suspected Cases of Viral Haemorrhagic Fevers in Nigeria

Olumuyiwa Babalola Salu[1,2]; Tomilola Feyikemi Akinbamiro[2]; Mercy Remilekun Orenolu[1]; Onyinye Dorothy Ishaya[2]; Roosevelt Amaobichukwu Anyanwu[1]; Olubunmi Rita Vitowanu[2]; Maryam Abiodun Abdullah[1], Adenike Hellen Olowoyeye[2]; Sodiq Olawale Tijani[2]; Kolawole Solomon Oyedeji[1,3] and Sunday Aremu Omilabu[1,2,]

[1] Centre for Human and Zoonotic Virology, Central Research Laboratory, College of Medicine of the University of Lagos, Idi-araba, Lagos, Nigeria.

[2]Department of Medical Microbiology and Parasitology, College of Medicine of the University of Lagos, Idi-Araba, Lagos, Nigeria.

[3]Department of Medical Laboratory Science, College of Medicine of the University of Lagos, Idi-Araba, Lagos, Nigeria.

Corresponding Author/Email: **Sunday Aremu Omilabu** (Professor of Medical Virology). Centre for Human and Zoonotic Virology, Central Research Laboratory and the Department of Medical Microbiology and Parasitology, College of Medicine of the University of Lagos, PM.B. 12003, Idi-Araba, Surulere, Lagos, Nigeria/Adjunct Professor, Nigerian Institute of Medical Research, P.M.B. 2013, Yaba, Lagos, Nigeria. Email: somilabu@unilag.edu.ng, omilabusa@yahoo.com. Mobile: +234 802 311 9431.

Abstract

There have been several Viral Hemorrhagic Fever (VHF) outbreaks in Nigeria which remains a public health concern. Despite the increasing number of suspected cases of VHF due to heightened surveillance activities and growing awareness, only a few cases are laboratory-confirmed to be VHF. Routinely, these samples are only tested for Lassa virus and Yellow fever virus with occasional testing for Dengue virus when indicated. The aetiology of the disease in these VHF suspected cases in Nigeria which are negative for Lassa, Yellow fever and Dengue viruses remains a puzzle. Since the clinical features exhibited by suspected VHF cases are like other endemic illnesses such as Hepatitis, there is a need to investigate the diversity and co-infections of hepatitis viruses as differentials and possible co-morbidity in suspected cases of VHFs in Nigeria. A total of three hundred and fifty (350) blood samples of 212 (60.6%) males and 138 (39.4%) females ages ranged <1 - 70 years and mean age of 25 ±14.5 suspected of VHFs were investigated for Hepatitis A, B, C and E viruses at the Centre for Human and Zoonotic Virology (CHAZVY), College of Medicine, University of Lagos (CMUL) using serologic and molecular techniques. The overall Enzyme Immunoassay Assay analysis revealed that 126 (36%) were positive for hepatitis. Individual prevalence revealed that none (0%) was reactive for HAV, while 37 (10.6%), 18 (5.1%) and 71 (20.3%) were positive for HBV, HCV and HEV respectively. A co-infection rate of 11.9% was also observed, with HCV/HEV co-infections being the most prevalent and the Northern region having the greatest burden of infection. The evidence of hepatitis virus infections in suspected cases of VHF was documented. Thus, their associations as co-morbidities and/or mortalities in this category of individuals require further investigations in endemic countries such as Nigeria. Therefore, the possible inclusion of screening for hepatitis viruses and other aetiologic agents that could mimic infections in suspected cases of VHFs in Nigeria should be thoroughly evaluated to guide informed policy on the diagnosis and management of these cases.

Keywords: Viral Haemorrhagic fever, Hepatitis A Virus, Hepatitis B Virus, Hepatitis C Virus, Hepatitis E Virus, Enzyme Immunoassay, Polymerase Chain Reaction.

Background/Introduction

Viral Haemorrhagic Fever (VHF) viruses are the major aetiologies of a group of diverse clinical syndromes. The manifestions of these infections range from asymptomatic to life threatening febrile illnesses with vascular damage with the possibility of haemorrhages in advanced cases [1-3]. However, the incidence of haemorrhage varies extensively between the different viruses [1,3]. Majorly, VHFs are caused by four taxonomic viral families of enveloped RNA viruses named: *Arenaviridae; Filoviridae; Flaviviridae; Bunyaviridae* [3-6]. The West African sub-region is endemic to several VHFs such as Lassa fever, Ebola haemorrhagic fever, Crimean-Congo haemorrhagic fever, Rift Valley fever, Dengue haemorrhagic fever and yellow fever [6-9]. Lassa virus the aetiology of Lassa fever is endemic in the countries of Guinea, Sierra Leone, Liberia, Mali, and Nigeria with most of these VHF entities having a zoonotic cycle with rodents and arthropods as the main reservoirs. These VHFs agents are transmitted to humans through contact with infected fluids from the reservoirs such as faecal matter, saliva, urine, other body fluids, and some may be transmitted through arthropods bites [3,8,9]. Person-to-person transmission with an incubation period for infection of about 2 - 21 days has also been documented [10]. The manifestations of the signs and symptoms of the diseases caused by these VHF agents shares similarities with most other endemic febrile illnesses in the West African sub-region such as malaria, hepatitis, influenza etc and differentiating between the agents of these illnesses remains a major challenge [10,11].

Viral hepatitis remains a major public health burden globally, driven by five unrelated hepatotropic viruses designated as caused as the hepatitis A virus (HAV), the hepatitis B virus (HBV), the hepatitis C virus (HCV), the hepatitis D virus (HDV), and the hepatitis E virus (HEV) [12,13]. These hepatitis agents has been documented to be associated with high disease burden, morbidity, mortality and has the potential to cause outbreaks and epidemics of about 50% of hepatitis cases worldwide [13-15]. Hepatitis A and E, causes hepatitis in both humans and various mammals and affects the liver like other hepatitis viruses [16-18]. Hepatitis A and E are typically transmitted faecal-orally by ingestion of contaminated food or water [19]. In majority of cases hepatitis A and E infections are asymptomatic or could be self-limiting with complete resolution within few weeks without treatment [20-23]. Globally, an estimated 101.7 and 28.4 million cases of HAV and HEV respectively has been documented as a result of sporadic cases and outbreaks reported mostly in resource limited settings [24]. In 2015, approximately 20 million people were reported to be infected by HEV with 44,000 deaths by the World Health Organization (WHO) [25]. These feacal-orally transmitted hepatitis agents

has major public heallth implications in Sub-Saharan Africa as HEV outbreaks are reported nearly annualy with some outbreaks involving more than 10,000 cases [12,16,26]. In Nigeria, there has been documented serologic evidence of previous exposure to HAV infection in most children by five years of age [5,12,27,28]. However, the actual burden and prevalences of both HAV and HEV are grossly underestimated in Africa due to some deficiencies in the surveillance and monitoring systems [29]. Transmission of Hepatitis B, C and D majorly occurs via contact with infected blood, blood products and other body fluids such as semen, vaginal fluids, and saliva [30]. Therefore, communal modes of spread are by transfusion of unscreened blood and its products, unprotected sexual activities, mother to child at birth, use of contaminated or inadequately sterilized instruments and sharing of sharp objects particularly during some traditional or cultural practices [31-33]. Individuals infected with these set of hepatitis viruses could develop chronic hepatitis, liver cirrhosis, and hepatocellular carcinoma with high morbidity and mortality rates if there are no interventions [15,30]. Globally, greater than 350 million people are chronically infected with HBV, while about 150 and 15 million are infected with HCV and HDV respectively [12,34,35]. Among those infected with HBV and HCV, Sub-Saharan Africa remains a behives of activities of these hepatitis viruses and Nigeria is one the most endemic countries within this region. The prevalence rates of both HBV and HCV ranges from 3 to 20% and 1 to 7% respectively within the general population [12,36].

Nigeria is endemic for both viral hepatitis and haemorrhagic fevers, but the actual interactions and disease burden of coinfections of these infectious agents is yet to be unravelled in the country. Over the decades, Nigeria have experienced yearly outbreaks of VHFs most especially Lassa fever disease with an increasing trend of morbidity and mortality in significant number of states since 2016 [10,37]. A surge in the number of suspected Lassa fever cases from 273 suspected cases in 2016 to 1022, 3498 and 3728 suspected cases in 2017, 2018 and 2019 respectively were documented in Nigeria with most of these suspected cases from Edo and Ondo states [2,38-40]. A similar trend of the increase in the case density and geographic distribution of Lassa fever across the states in Nigeria from 2018 to 2020 has also being documented by Dalhat et al., 2022 [41]. Despite the increasing trends of Lassa fever suspected cases in Nigeria, Chikungunya and West Nile viruses have also been detected in febrile patients in Ile-Ife, Osun state [42], while Dengue was reported from patients in Ibadan [43] and Ilorin [44]. However, amongst the VHF suspected cases in Nigeria, only few cases are laboratory confirmed with majority of cases unconfirmed for any aetiologic agent in Nigeria. Therefore, this study aims to investigate the detection of hepatitis viruses (HAV, HBV, HCV and HEV)

as co-morbidity and evaluate the different patterns of co-infections in suspected but unconfirmed cases of viral haemorrhagic fever cases in Nigeria.

Materials and Methods

Study Design/Location/Population: This was a retrospective cross-sectional study carried out at the Centre for Human and Zoonotic Virology (CHAZVY), College of Medicine of the University of Lagos (CMUL), Idi-Araba, Lagos, Nigeria between January, and March 2022. A total of three hundred and fifty (350) samples confirmed negative by Reverse Transcriptase Polymerase Chain Reaction (RT-PCR) for Lassa virus, Yellow fever, and Dengue virus respectively from individuals aged between <1 and 70 years were analyzed in this study. These samples were retrieved from the -80°C sample bank facility at CHAZVY of suspected cases of VHF samples received between 2017 and 2020 from Lassa fever treatment centers across the country.

Ethical Consideration: Ethical approval for this study with exemption from informed consent was received from the Health Research Ethics Committee (HREC), College of Medicine of the University of Lagos (CMUL) with the approval number: CMUL/HREC/02/20/718. Fully anonymized confidential data were accessed, kept confidential, stored in accessed protected laptops and analyzed as part of VHF public health response in Nigeria of stored samples at the Centre for Human and Zoonotic Virology (CHAZVY) with permission from the laboratory management.

Data/Specimen Retrieval and Storage: Anonymized laboratory unique identifier codes for negative plasma samples of suspected VHF cases from various locations stored at CHAZVY randomly selected by the data manager were accessed between Monday 8th to Friday 12th June 2020. Corresponding plasma samples of the selected codes were sorted from the -80^{0}C storage freezers using the Universal precautions as recommended by the Centre for Diseases Control and Prevention [45,46]. The cold chain of the samples was maintained, and randomly selected plasma samples were returned to the -80°C freezer until analysis.

Serological Analysis using Enzyme Linked Immunosorbent Assay (ELISA): All selected stored plasma samples were analyzed and interpreted according to manufacturer's instructions (Aria, CTK Biotech Inc, USA) for the detection of antibodies for Hepatitis A Virus Immunoglobulin M (HAV IgM), Hepatitis C Virus Antibody (HCV Ab) and Hepatitis E Virus

Immunoglobulin M (HEV IgM) and detection of antigen for Hepatitis B Virus Surface Antigen (HBsAg) serologically.

Nucleic acid extraction and Polymerase Chain Reaction (PCR): viral genetic materials from selected sample aliquots were extracted according to manufacturer's instructions in a biosafety level 2 laboratory using a mini spin column RNA and DNA extraction kits (Qiagen, Germantown, Maryland, United States) respectively. The extracted genetic materials (DNA/RNA) samples were used for Reverse Transcription PCR (RT-PCR) and PCR amplification respectively using the primer sets highlighted in table 1. For HAV, HCV and HEV, 5 µL of the extracted RNA was amplified in 20ul of RT-PCR master mix reagents (JENA HOT START MIX) prepared using 5 µL of master mix buffer, 1µL each of forward and reverse primers (Table 1), and 13 µL of Nuclease-free water. The amplification was done using the Applied Biosystems Thermocycler with the following cycling conditions; 30mins at 50^0C, 5mins at 95^0C, followed by 35 cycles of 15 secs at 95^0C, 15secs at 55^0C, 20secs at 68^0C and 3mins at 68^0C for final elongation. While for HBV, 3 µL of the extracted DNA was amplified 22 µL master mix prepared using 5 µL of master mix buffer, 1 µL each of forward and reverse primers (Table 1) and 15 µL of Nuclease-free water. The PCR cycling conditions were 94^0C for 5mins followed by 40 cycles of 30 secs at 94^0C, 30secs at 55^0C, 20secs at 72^0C and final elongation at 72^0C for 5mins. The generated PCR amplicons were analyzed using 2.5% agarose gel electrophoresis with 1X SYBR® safe DNA gel staining dye (Invitrogen, Carlsbad, California, United States) run at 120V/400mA for 30 minutes. The resolved amplicon bands images were captured with the BioDocAnalyze 2.0 (Biometra, Goettingen, Germany) under UV light and stored on restriction access computer system within CHAZVY.

Results

A total of 350 retrieved samples from suspected VHF cases collected between 2017 and 2020 at CHAZVY were screened for HAV and HEV IgM, HBsAg and anti-HCV by ELISA. The participants include 212 (60.6%) males and 138 (39.4%) females with a ratio of 1:1.5 and majority (92.0%) are alive (Table 2). Their age ranged from <1 - 70 years, and the mean age of all participants was 25 ±14.5 years. The age range with the highest number of participants was 21-30 years with 98 (28.0%) participants, followed by 11-20 years with 71 (20.3%) and the range with the least number of participants is ≤1 with 4 (1.1%) (Table 2). Majority of these suspected cases samples were sent from the South-west, North-east and North-west of Nigeria

with frequency rates of 35.1%, 34.3% and 15.4% respectively and the most documented symptom was fever (87.4%) with 12.6% having both fever and bleeding (Table 2).

Out of the 350 samples analyzed, 126 (36%) of these VHF suspected cases were positive for at least one of the hepatitis viruses by ELISA (Table 3). Among the 126 (36%) VHF suspected cases that were positive for hepatitis viruses, the distribution of the specific hepatitis virus (single infection) detected revealed that none (0%) was reactive for HAV, while 37 (10.6%), 18 (5.1%) and 71 (20.3%) were positive for HBV, HCV and HEV respectively (Table 3). Further evaluation of coinfections of hepatitis viruses in these cohorts of positive individuals showed an overall coinfection of 15 (11.9%). Dual coinfections of HBV/HCV, HBV/HEV, HCV/HEV and triple coinfections of HBV/HCV/HEV were observed with prevalence rates of 3 (2.4%), 2 (1.6%), 8 (6.3%) and 2 (1.6%) respectively (Table 2). Majority (62.7%) of those positive for at least one hepatitis virus were males compared to their female's counterpart who had a prevalence of 37.3%. Most of the positive hepatitis samples were within age groups 21-30, 11 – 20 and 31-40 years with prevalence rates of 36 (10.3%), 31 (8.9%) and 21 (6%) respectively (Table 3). The positivity rates of at least one hepatitis virus detected based on the geographic regions of Nigeria showed that the North-East region has the highest number of positives with 67 (19.1%), North-West region with 23 (6.6%) and South-West region with 22 (6.2%) positives. While the South-South and North-Central both had 7 (2%) positive rates respectively. However, no positive case was detected among samples from South-East region (Table 3).

Comparative analysis of the distribution of hepatitis positive samples of the geographic regions with dual and triple coinfections showed that out of 15 positive samples with evidence of coinfection, majority (40%) were detected from North-East region with an even distribution (50%) between the male and female (Table 4). Twenty percent (20%) each were positive from the North-West, South-West and South-South regions respectively, while there was no (0%) coinfection of hepatitis viruses among suspected cases from the North-Central and South-East regions (Table 4). Similar male to female positive ratios of 2 to 1 were observed in the North-West and South-West regions, while a reverse ratio of 2 to 1 female to male positive was documented for the South-South region (Table 4).

The reverse transcriptase polymerase chain reaction (RT-PCR) investigation carried out on the 126 samples which tested positive for at least one hepatitis virus by Enzyme Immunoassay revealed the presence of expected amplicon band sizes base pairs (bp) as confirmation for HBV

(860bp), HCV (187bp) and HEV (171bp) respectively as highlighted in figure 1, 2, and 3. The expected amplicon band sizes for HBV, HCV and HEV genetic materials were as compared with their positive controls bands as highlighted on the gel image (Figure 1, 2, and 3). The negative control lane had no visible bands (Figure 1, 2, and 3). However, no amplicon band was observed for the expected band sizes (192 bp) for HAV (Figure 4).

Discussion

The resource limited countries particularly in sub-Saharan Africa remains a beehive of febrile illnesses with multiple etiological agents which are difficult to distinguish based on the similarities of their signs and symptoms. The manifestation of febrile illnesses could be because of parasitic diseases such as Malaria, Toxoplasmosis, Schistosomiasis; bacteria diseases such as Typhoid, Typhus, Borreliosis, Leptospirosis; or by viral diseases such as Cytomegalovirus, Mumps, Measles, Rubella, Coxsackie, Yellow fever, Hepatitis, Lassa, Marburg, Ebola, Dengue, Crimean–Congo and Hantaan viral haemorrhagic fevers [3,5,8,9,28,47-49]. Over the decades, these diseases have been major public health problems in the sub-Saharan African region with limited or no information on the actual burdens of these agents and their interactions as co-morbidities and mortalities in febrile illnesses. A better understanding of the in-country epidemiology of these febrile illnesses' agents could evidence their possible inclusion as differential diagnoses in suspected cases of VHF [5,47]. Nigeria had witnessed a surge in the number of states reporting suspected cases of VHF since 2016 [10,41]. Viral hepatitis and VHFs infections are two public health problems with endemicity in-country and there are difficulties in distinguishing VHFs from other endemic febrile agents circulating in Nigeria and other African countries. Considering that only limited number of VHF agents are routinely tested for in Nigeria and the dearth of unswerving epidemiological data on the contributions of other aetiologic agents that present with similar symptoms as VHFs, the contribution of hepatitis viruses as differential causative agents, co-morbidities, and mortalities in suspected but unconfirmed cases of VHFs in Nigeria was elucidated in this study.

Findings from this study suggests that hepatitis virus infections should be considered as differential aetiologic agents that might be a major comorbidity in the outbreaks of viral haemorrhagic fevers in Nigeria as 36% of these samples suspected of VHF but confirmed to be negative for Lassa, Yellow fever and Dengue viruses were reactive for at least one hepatitis virus. The association of these hepatitis viruses with other VHF agents might be crucial in the

differential diagnosis and management of these endemic diseases within our environment. These agents have been documented to possess similar clinical symptoms such as renal failure, hepatic injury, jaundice, fever, fatigue, loss of appetite, nausea, or vomiting [50-52]. Thus, effects of these viral hepatitis agents on the burden of VHFs in Nigeria cannot be ruled out and as such it is methodical to diagnose other viral hepatitis to determine its effect on VHFs on routine basis [1,48]. Several studies have documented coinfections of some hepatitis virus in suspected cases of VHFs in some parts of Africa such as HAV and HCV coinfections as well as in Dengue and yellow fever infections but not in Nigeria [5,28,47,48,53,54]. This present study provides documented evidence of the co-circulation and the burden of hepatitis virus in the cohort of suspected but unconfirmed cases of VHFs in Nigeria. Coinfections of these hepatitis viruses, particularly in endemic countries, should be further investigated since the pathway of liver involvement and degeneration in VHF cases has not been fully elucidated [28,54]. In endemic areas, the knowledge of these hepatitis and other viruses that might be co-circulating in suspected cases of VHFs can help to make proper differential diagnoses and to apply adequate diagnostic methods. Therefore, it might be reasonable to screen for these viruses in suspected cases of VHFs since signs and symptoms are superimposed on each other which could lead to missed diagnosis [1,3,5].

Based on the individual hepatitis virus detected among these suspected cases of VHFs in Nigeria, data from this study revealed HEV infections as the most prevalent (20.3%), followed by HBV and HCV with prevalence rates of 10.6% and 5.1% respectively. None was positive for HAV infection in these study population. These findings further confirmed the endemicity of these hepatitis viruses within our environment. However, it is not surprising that none of these individuals was positive for Hepatitis A Virus IgM in an endemic country such as Nigeria. This might be connected to the fact that, nearly all children might have encountered HAV at early age with asymptomatic manifestations, expression of serological evidence of prior exposure and within the development of long-term immunity [12,33,55-57]. To collaborate this finding a demographic study among children within the Kaduna metropolis in Nigeria also reported very low prevalence rate of 0.67% among 300 participants [58]. This might be due to the downward trend in HAV cases in the country. The detection rates of HAV and HEV were not comparable in this study, despite that both viruses are transmitted through contaminated water and food sources [59,60,61]. The detected HEV prevalence was statistically significant in this study and shows there is high activities of the virus in Nigeria. This suggests that the endemicity of this agent among suspected cases of VHFs in our environment requires extensive

evaluations. Thus, HEV might be a major additional disease burden fuelling the increasing numbers of suspected cases of VHFs as reported from known endemic and non-endemic states recently in Nigeria. The evidence of HEV as documented here was significantly higher than the 0.4% obtained among pregnant women in Oyo and Anambra communities [62], 0.9% among different populations in Plateau State [63], and the 2.7% in apparently healthy participants in Ogbomoso, South-western Nigeria [64]. However, the finding from this study was like the 17.8% seroprevalence rate obtained from a study in Lagos [65].

The detected HBV and HCV prevalence in this study was at a ratio of 2:1, showing that HBV was twice as likely to be more prevalent in this category of population in our environment. This finding supports the documented evidence that globally, over 350 and 150 million people are chronically infected with HBV and HCV respectively [12,36]. This data also confirms the endemicity and wide spread of both viruses in Sub-Saharan Africa including Nigeria with common transmission routes. The highest prevalence rates of between 3 – 20% and 1 – 7% for both HBV and HCV respectively have also been documented to be domicile among the general population within the sub-Saharan Africa region [12]. Although, varied rates for both HBV and HCV prevalence have been documented in Nigerian population with an estimated/pooled 14% and 2.1% prevalence rates of Hepatitis B virus and Hepatitis C infections respectively [66-68]. The prevalence for HBV and HCV documented in this study population might have significant consequences on the management of suspected cases of VHF as both viruses remain silent killers within our environment. Co-infection patterns observed highlight that HCV/HEV co-infection was mostly prevalent which was even higher than that of HBV/HCV coinfection which was surprising but not statistically significant in this study. However, risk factors such as blood transfusion, scarification, circumcision, and heterosexual activities that might be associated with these coinfections among these suspected cases that were negative for any VHF agents in Nigeria were not evaluated in this study. The lower coinfection rates of HBV with other hepatitis viruses in this study could be due to the increased public awareness programmes and improved vaccination uptakes reported within the country. More follow ups regarding HBV awareness have been done with routine investigations carried out during blood screenings and even in pregnant women. Also, business owners and industries including government establishments have mandated HBV vaccination for their staffs.

The Northern region especially the North-East recorded the highest prevalence of both single and hepatitis virus co-infection amongst these cohort of suspected cases of VHF in Nigeria particularly for HEV infections. This finding from the North-East might not be unconnected to

various risk factors such as insecurity, vulnerability of children, level of hygiene, level of contamination, overpopulation, poor electricity supply, poor water supply [58,63,69]. This part of the country had witnessed high level of insurgencies in recent pasts with majority of people being displaced from their settlements. Many of these displaced population reside now in makeshift's internally displaced camps with limited hygienic conditions. These camps have various issues with limited or no facility for toilets, sewage disposal and no hand washing station. Children born within the North-Eastern part of Nigeria might not be properly cared for by parents due to the insurgency issues with no possibility for proper education. Many of these parents leave their ward to roam and source or beg for food and money in their tender age, and feed on anything that comes their way daily without minding how clean, or good the food they are consuming is. Also, the detection rates in this part of the country might not be unconnected with other factors that might be drivers for these infections including, the hygiene level in the rural settings that is so poor as they drink from rivers where they wash and have their baths; they do not wash or sanitize hands before feeding even when the palms are so dirty; they do not store their meals properly as some return to uncovered foods with houseflies on them [63,68,69]. Furthermore, this part of Nigeria is largely occupied by a certain religious group, with religious believes permitting having more than one wife and having as many children as they desire. Aside this, most of the girl child are married off after their first menstrual cycle which is usually between age 11-15, to start procreating. Water supply systems provided in these parts is insufficient and occupant's search for other means such as the rivers water that are not purified to drink and cook their meals.

The highest frequencies of at least a single hepatitis infection and coinfections were documented amongst age groups 11 – 20 and 21 – 30 years respectively in this study population. These are a very active group and most of them want unhindered freedom. They engage in lots of activities which could be social or environmental, some of them even care about their social status and outward look than their health status. At this age, individuals feel caged and controlled therefore want their freedom, this leads to corrupt and immoral practices. They get involved in acts and practices that could lead to hepatitis a lot, such as smoking, drinking, drug abuse, drawing of tattoos underneath the skin, pre-marital sex amongst others which are cases of HBV and HCV infections. The findings in these age groups agrees with other studies of HEV infections in Nigeria [64,70], but was at variance with the report of Ifeorah *et al*., [62]. This age group often eat out for convenience's sake without caring about hygiene levels of eateries that they patronize. Thus, they might be prone to ingestion of

contaminated food and water. A higher prevalence of HEV in males as compared to females as seen in this study was comparable to the studies by Buti *et al*., in 2008 [71]; and Oladipo *et al*., in 2017 [64] but was at variance with the report Fowotade *et al*., in 2018 [70]. Generally, observations have shown that the female population have a better hygiene level compared to the male population, this also could be a reason for the higher numbers of affected males to females.

Conclusion

The evidence of hepatitis virus infections in suspected cases of viral hemorrhagic fever in Nigeria as documented in this study suggest the inclusion of differential diagnosis for hepatitis viruses among these cohort of individuals. The study also shows that there might be possible co-morbidity between viral hemorrhagic fevers and hepatitis viruses although there was not statistically significance correlation with the co-infection of hepatitis A, B and C except for HEV which was statistically significant based on the findings from this study. Thus, their associations as co-morbidities and mortalities in this category of individuals requires further investigations in endemic countries such as Nigeria. Therefore, the possible inclusion of screening platforms for hepatitis viruses and other aetiologic agents that could mimic infections in suspected cases of VHFs in Nigeria should be thoroughly evaluated to guide informed policy on the diagnosis and management of these cases.

Financial Support and Sponsorship

No financial support was received by the Centre for Human and Zoonotic Virology (CHAZVY), College of Medicine, University of Lagos, Lagos, Nigeria for the execution of this study.

Conflicts of Interest

The Authors declares no conflicts of interest regarding this study.

Authors Contribution

Conceptualization and design of study: Salu OB, Omilabu SA.

Data extraction and storage: Orenolu MR, Anyanwu RA, Abdullah MA, Akinbamiro TF, Ishaya OD, Vitowanu OR, Olowoyeye AH, Tijani SO, Oyedeji KS.

Analysis of samples: Orenolu MR, Anyanwu RA, Abdullah MA, Akinbamiro TF, Ishaya OD, Vitowanu OR, Olowoyeye AH, Tijani SO

Data analysis: Salu OB, Omilabu SA, Orenolu MR, Anyanwu RA, Abdullah MA, Akinbamiro TF, Ishaya OD, Vitowanu OR, Olowoyeye AH, Tijani SO, Oyedeji KS.

Writing – original draft: Salu OB, Ishaya OD.

Writing – review and editing: Salu OB, Omilabu SA, Orenolu MR, Anyanwu RA, Abdullah MA, Akinbamiro TF, Ishaya OD, Vitowanu OR, Olowoyeye AH, Tijani SO, Oyedeji KS.

Acknowledgment: The authors acknowledge and thank the data officer of the Centre for Human and Zoonotic Virology (CHAZVY), College of Medicine, University of Lagos (CMUL) for assisting with the extraction of data.

References

1. Bonney, J. H. K., Osei-Kwasi, M., Adiku, T. K., Barnor, J. S., Amesiya, R., Kubio, C. and Günther, S. (2013). Hospital-Based Surveillance for Viral Hemorrhagic Fevers and Hepatitides in Ghana. *PLoS Neglected Tropical Diseases, 7(9), e2435.* doi: 10.1371/journal.pntd.0002435
2. Nigeria Centre for Disease Control (NCDC) 2017. National Guidelines on Infection Prevention and Control of Viral Hemorrhagic Fevers.
3. van Leeuwen LPM, de Jong W, Doornekamp L, van Gorp ECM, Wismans PJ, Goeijenbier M. Exotic viral hepatitis: A review on epidemiology, pathogenesis, and treatment. J Hepatol. 2022 Nov;77(5):1431-1443. doi: 10.1016/j.jhep.2022.06.031. Epub 2022 Jul 9. PMID: 35817222.
4. Racsa D. Lori, Colleen S. Kraft, Gene G. Olinger and Lisa E. Hensley (2016). Viral Haemorrhagic Fever Diagnostics. *Clinical Infectious Diseases, 62(2) 214-9.* doi10.1093/cid/civ792.
5. Alemad SA, Halboup AM, Aladeeb K, Al-Saleh M, Al-Kufiley N. Coinfection with dengue and hepatitis A complicated with infective endocarditis in a Yemeni patient: a case report. J Med Case Rep. 2021 Sep 11;15(1):450. doi: 10.1186/s13256-021-03069-w. PMID: 34507614; PMCID: PMC8434717.
6. Cobo F. Viruses Causing Hemorrhagic Fever. Safety Laboratory Procedures. Open Virol J. 2016 Jan 15; 10:1 - 9. doi: 10.2174/1874357901610010001. PMID: 27014378; PMCID: PMC4780467.
7. Ftika L, and Maltezou HC. (2013). Viral haemorrhagic fevers in healthcare settings. *J Hosp Infect* 83, 185-192.
8. Chen, L.H.; Wilson, M.E. Yellow fever control: Current epidemiology and vaccination strategies. Trop. Dis. Travel Med. Vaccines 2020, 6, 1–10.
9. Diagne, M.; Ndione, M.; Gaye, A.; Barry, M.; Diallo, D.; Diallo, A.; Mwakibete, L.; Diop, M.; Ndiaye, E.; Ahyong, V.; et al. Yellow Fever Outbreak in Eastern Senegal, 2020–2021. Viruses 2021, 13, 1475.
10. Salu O.B, James A.B., Orenolu M.R, Anyawu R.A, Abdullal I.J., Abdus-Salam I.A., Ihekweazu C. and Omilabu S.A (2019). Lassa virus RNA detection from suspected cases in Nigeria, 2011-2017. *The Pan African Medical Journal* 6:34:76.
11. Raabe V, and Koehler J. Laboratory diagnosis of Lassa fever. Journal of Clinical Microbiology. 2017; 55(6): 1629 –1637. https://doi.org/10.1128/JCM.00170-17.

12. Makiala-Mandanda S, LeGa lF, Ngwaka-Matsung N, Ahuka-Mundeke S, Onanga R, Bivigou-Mboumba B, Pukuta-Simbu E, Gerber A, Abbate JL, Mwamba D, Berthet N, Leroy EM, Muyembe-Tamfum J-J, Becquart P. 2017. High prevalence and diversity of hepatitis viruses in suspected cases of yellow fever in the Democratic Republic of Congo. J Clin Microbiol 55:1299 – 1312. https://doi.org/ 10.1128/JCM. 01847 - 16.
13. World Health Organization (2020). Hepatitis E. Key facts. *Who.int/news.room/factsheets/detail/hepatitis E.*
14. Omote V, Kashibu E, Ojumah I, Adda D, Etaghene J, Ukwamedua H. (2018). Serological screening of hepatitis B virus and hepatitis C virus among patients attending a tertiary hospital in Jalingo, Taraba state, Nigeria. *Saudi Journal Health Sci* 2018; 7:167-71. doi: 10.4103/sjhs.sjhs_39_18
15. World Health Organisation, (2019). What is hepatitis? An online question and answer reviewed September 2019.
16. Kim JH, Nelson KE, Panzner U, Kasture Y, Labrique AB, Wierzba TF. 2014. A systematic review of the epidemiology of hepatitis E virus in Africa. BMC Infect Dis 14:308. https://doi.org/10.1186/1471-2334-14-308.
17. Joon A, Rao P, Shenoy SM, Baliga S. 2015. Prevalence of hepatitis A virus (HAV) and hepatitis E virus (HEV) in the patients presenting with acute viral hepatitis. Indian J Med Microbiol 33 Suppl:102–105.
18. Reem, A.A., Awatif, N.A., Salma, A.A., Jawad, U.R., Basavaraj, C.H. and Khaled, R.A. (2021). Prevalence of Hepatitis E Virus infection among Blood donors in the Eastern Province of Saudi Arabia. *Journal of Multidisciplinary Healthcare*. 2381-2390.
19. Aggarwal, R., and Goel, A., (2016). Advances in hepatitis E - I: virology, pathogenesis, and diagnosis. *Expert Review Gastroenterology Hepatology*; 10:1053–1063.
20. Tedder RS, Ijaz S, Kitchen A, Ushiro-Lumb I, Tettmar KI, Hewitt P, Andrews N. Hepatitis E risks: pigs or blood-that is the question. Transfusion. 2017; 57:267–72.
21. Niederhauser C, Widmer N, Hotz M, Tinguely C, Fontana S, Allemann G, Borri M, Infanti L, Sarraj A, Sigle J, et al. Current hepatitis E virus seroprevalence in Swiss blood donors and apparent decline from 1997 to 2016. Euro Surveill. 2018; 23:1700616.
22. Bader, Y.A., Waleed, A.A., Mohammed, S.A., Sami, M.A., Rayan, N.A., Abeer, S.A., Sulaiman,H.A., Ahmed, A.A., Ammar, H.A., Yara, E.B. and Abdulrahman, A.A. (2019). Seroprevalence of Hepatitis E viruses among Blood Donor in the Qassim Region, Saudi Arabia.
23. Tsoi WC, Zhu X, To AP, Holmberg J. Hepatitis E virus infection in Hong Kong blood donors. Vox Sang. 2020; 115:11–7.
24. Kmush B, Wierzba T, Krain L, Nelson K, Labrique AB. 2013. Epidemiology of hepatitis E in low- and middle-income countries of Asia and Africa. Semin Liver Dis 33:15–29. https://doi.org/10.1055/s-0033-1338111.
25. Fu P, Lin B, Wu B, Ke L, Yang T, Du Y, Cheng L, Li Z, Li T, Liu Y. Hepatitis E virus prevalence among blood donors in Dali, China. Virol J. 2021 Jul 7;18(1):141. doi: 10.1186/s12985-021-01607-y. PMID: 34233712; PMCID: PMC8261953.
26. Spearman CW, and Sonderup MW. 2015. Health disparities in liver disease in sub-Saharan Africa. Liver Int 35:2063–2071. https://doi.org/10.1111/liv .12884.
27. Jacobsen KH. 2014. Hepatitis A virus in West Africa: is an epidemiological transition beginning? Niger Med J 55:279–284. https://doi.org/10.4103/ 0300-1652.137185.

28. Ranathunga .I ,1 L. S. Kannangara,2 K. A. S Bandara,1 and S. B. Gunatilake (2019). A Rare Presentation of Coinfection: Dengue Virus and Hepatitis A Virus. Case Reports in Infectious Diseases Volume 2019, Article ID 9782892, 2 pages https://doi.org/10.1155/2019/9782892
29. Gerbi GB, Williams R, Bakamutumaho B, Liu S, Downing R, Drobeniuc J, Kamili S, Xu F, Holmberg SD, Teshale EH. 2015. Hepatitis E as a cause of acute jaundice syndrome in northern Uganda, 2010–2012. Am J Trop Med Hyg 92:411–414. https://doi.org/10.4269/ajtmh.14-0196.
30. Thursz M, and Fontanet A. 2014. HCV transmission in industrialized countries and resource-constrained areas. Nat Rev Gastroenterol Hepatol 11:28–35. https://doi.org/10.1038/nrgastro.2013.179.
31. *World Health Organization (2014). "Ebola virus disease Fact sheet No. 103"; September 2014.*
32. Adebola T. Olayinka, Akin Oyemakinde, Muhammad S. Balogun, Anthonia Ajudua, Patrick Nguku, Moses Aderinola, Abiodun Egwuenu-Oladejo, Simeon W. Ajisegiri, Samuel Sha'aibu, Bolanle O. P. Musa, Saheed Gidado,1 and Abdulsalami Nasidi (2016). *American Journal of Tropical Medicine and Hygiene*, 95(4), 2016, pp. 902–907doi:10.4269/ajtmh.15-0874
33. Jefferies, M., Rauff, B., Rashid, H., Lam T. and Rafiq S. (2018). Update on global epidemiology of viral hepatitis and preventive strategies. *World Journal of Clinical Cases*, 6(13): 589-599. doi: http://dx.doi.org/10.12998/wjcc.v6.i13.589.
34. Centers for Disease Control and Prevention. (2020). *Health disparities.* Centers for Disease Control and Prevention, Division of Adolescent and School Health, National Center for HIV/AIDS, Viral Hepatitis, STD, and TB Prevention. https://www.cdc.gov/healthyyouth/disparities/index.htm
35. Akande, E. B., Oladejo B. O., Oladunmoye M. K., Abodunrin T. F., and Ogunlana, D.O. Prevalence Of Hepatitis C Positivity In Apparently Healthy Individuals In Akure, Nigeria. Nat Sci 2020;18(6):9-16]. ISSN 1545-0740 (print); ISSN 2375-7167 (online). http://www.sciencepub.net/nature. 3. doi:10.7537/marsnsj180620.03
36. Rao VB, Johari N, du Cros P, Messina J, Ford N, Cooke GS. 2015. Hepatitis C seroprevalence and HIV co-infection in sub-Saharan Africa: a systematic review and meta-analysis. Lancet Infect Dis 15:819–824. https://doi .org/10.1016/S1473-3099(15)00006-7.
37. Mofolorunsho KC. 2019. Outbreak of lassa fever in Nigeria: measures for prevention and control. Pan African Medical Journal. 23 (1).
38. Mustapha A. Lassa fever: Unveiling the misery of the Nigerian health worker. Ann Nigerian Med [serial online] 2017 [cited 2020 Feb 10]; 11:1-5. Available from: http://www.anmjournal.com/text.asp?2017/11/1/1/225608.
39. Amodu SE and Fapohunda. Lassa fever and the Nigerian experience: a review European Journal of Biological Research 2019; 9(3): 155-164. http://dx.doi.org/10.5281/zenodo.3382695.
40. Nigeria Centre for Disease Control (NCDC) 2019. An update of lassa fever outbreak in Nigeria. [Accessed 1st November,2019]. https://ncdc.gov.ng›diseases›sitreps.
41. Dalhat MM, Olayinka A, Meremikwu MM, Dan-Nwafor C, Iniobong A, Ntoimo LF, et al. (2022) Epidemiological trends of Lassa fever in Nigeria, 2018–2021. PLoS ONE 17(12): e0279467. https:// doi.org/10.1371/journal.pone.0279467
42. Adesina OA, Japhet MO, Omilabu SA. Detection of Chikungunya and West Nile viruses in febrile patients in Ile-Ife Osun State, Nigeria using real time reverse

transcription-polymerase chain reaction (RT-PCR). African Journal of Microbiology Research. 2017 Jul 28;11(28):1136-41.
43. Onoja AB, Adeniji JA, Olaleye OD. High rate of unrecognized dengue virus infection in parts of the rainforest region of Nigeria. Acta tropica. 2016 Aug 1;160: 39-43.
44. Adedayo F, Nioma I, Olanrewaju MB, Adeyinka A, Ebele A. Serological evidence of recent dengue virus infection among febrile children in a semi-arid zone. American Journal of Infectious Diseases. 2013 Jan 1;9(1):7.
45. Nigeria Centre for Disease Control, (2017). National Guidelines on Tnfection Prevention and Control of Viral Haemorrhagic Fevers. *NCDC Guidebook* 1-60.
46. Center for Disease Control (2016). Prevention/Isolation precaution/ Guideline. cdc.gov/prevention
47. Machain-Williams C, Talavera-Aguilar L, Cetina-Trejo R.C, Carrillo-Navarrete J, Rivero-Cárdenas N, Salazar M.I, Farfán-Ale J.A, Puerto F.I, and Castro-Mussot M.E. (2014). Detection of Hepatitis C Virus Coinfection in Patients with Dengue Diagnosis**.**
48. Gadia C.LB, Manirakiza A, Tekpa E, Konamina X, Vickos U and Nakoune E (2017). Identification of pathogens for differential diagnosis of fever with jaundice in the Central African Republic: A retrospective assessment, 2008-2010. *BMC Infectious Diseases* 17: 735-740.
49. Yandai, F.H.; Traore, K.A.; Moussa, A.M.; Ouoba, B.L.; Ouoba, J.B.; Bolti, M.A.; Abakar, M.F.; Hota, M.; Gamougam, K.; Nadlao, B.; et al. Prevalence and Diversity of Hepatitis Virus Markers among Patients with Acute Febrile Jaundice in Chad. Microbiol. Res. 2021, 12, 878–887. https://doi.org/10.3390/microbiolres 12040064
50. Madani TA, Al-Mazrou YY, Al-Jeffri MH, Mishkhas AA, Al-Rabeah AM, et al. (2003) Rift Valley fever epidemic in Saudi Arabia: epidemiological, clinical, and laboratory characteristics. *Clin Infect Dis* 37: 1084–1092
51. Tomori, O. (2004). Yellow fever: the recurring plague. *Critical Reviews in Clinical Laboratory Sciences*, 41: 391–427.
52. Asogun, D.A., Adomeh, D.I., Ehimuan, J., Odia, I., Hass, M., Gabriel, M., Olschlager, S., Becker-Ziaja, B., Folarin, O., Phelan, E., Ehiane, P.E., Ifeh, V.E., Uyigue E.A., Oladapo Y.T., Muoebonam, E.B., Osunde, O., Dongo A., Okokhere P.O., Okogbenin S.A, Momoh M., Alikah, S.O., Akhuemokhan O.C., Imomeh, P., Odike M.A.C., Gire S., Andersen K., Sabeti P.C., Happi C.T., Akpede, G.O., Gunther, S., (2012) Molecular diagnostics for lassa fever at Irrua specialist teaching hospital, Nigeria: lessons learnt from two years of laboratory operation. *PLoS Neglected Tropical Disease* 6(9): e1839.
53. Zaki, A.S and Lad, V. (2011) Concurrent infection of Dengue fever and Hepatitis A infection: A case report. Indian J. Crit. Care Med.; 15 (4): 238-240.
54. Seneviratne SL, Malavige GN, de Silva HJ. Pathogenesis of liver involvement during dengue viral infections. Trans R Soc Trop Med Hyg 2006;100(7):608–614.
55. World Health Organization (WHO, 2010). The global prevalence of hepatitis A virus infection and susceptibility: a systematic review. Geneva: World Health Organization.
56. Franco E, Meleleo C, Serino L, Sorbara D, Zaratti L. (2012). Hepatitis A: Epidemiology and prevention in developing countries. *World Journal of Hepatology*, 4: 68-73 (PMID: 22489258 DOI: 10.42548).
57. Hutin, Y., Low-Beer, D., Bergeri, I., Hess, S., Garcia-Calleja, J.M., Hayashi, C., Mozalevskis, A., Rinder, S.A., Sabin, K., Harmanci, H. Bulterys, M. (2017). Viral

Hepatitis Strategic Information to Achieve Elimination by 2030: Key Elements for HIV Program Managers. *JMIR Public Health and Surveillance*, 3: e91 (PMID: 29246882 DOI: 10.2196/publichealth.7370) ID 9782892, https://doi.org/10.1155/2019/9782892.

58. Bugaje, MA, Balogun SE, Abdulkabir, I, Ahmed AA. (2011). Seroprevalence of Hepatitis E infection among secondary school students in Kaduna State, Northern Nigeria. Sahel Medical Journal. 14(3): 143-147
59. Peter, E. and Mark, R. (2016) Leveraging urbanization in South Asia, managing spatial transformation for prosperity and livability. Washington, DC: World Bank. Https://openknowledge.worldbank.org/handle/10986/22549.
60. Rein, D.B., Stevens, G.A., Theaker, J., Wittenborn, J.S. and Wiersma, S.T. (2012). The global burden of hepatitis E virus genotypes 1 and 2 in 2005. *Hepatology*, 55: 988-997 (PMID: 22121109 DOI: 10.1002/ hep.25505).
61. Nimgaonkar I, Ding Q, Schwartz RE, Ploss A. Hepatitis E virus: advances and challenges. Nat Rev Gastroenterol Hepatol.2018;15(2):96-110
62. Ifeorah I.M, Faleye T.O.C, Bakarey A.S, Adewumi M.O, Akere A, Omoruyi E.C, Ogunwale A.O and Adeniji J.A (2017). Acute Hepatitis E virus infection in two geographical regions of Nigeria. *Hindawi Journal of pathogens* 10:1-6.
63. Junaid SA, Agina SE, Abubakar KA. Epidemiology and associated risk factors of hepatitis E virus infection in Plateau State, Nigeria. Virology. 2014; 5: 15-26. https://doi.org/10.4137/VRT.S15422 PMid:25512696 PMCid: PMC4251053
64. Oladipo, E.K, Oloke J.K, Awoyelu E.H, Olasunmibo O.G, Ogunyale T.T (2017). Detection of Hepatitis E virus among HIV infected individuals in Ogbomoso, South-western Nigeria. *British Journal of Virology* 2(4): 62-67.
65. Meseko C, Odurinde O, Odaibo G and Olaleye D (2016). Serological evidence and risk factors associated with hepatitis E virus infection in pigs and human at an intensive piggery complex, Lagos Nigeria. *Journal of Healthcare Communication* 1: 1.
66. Ikobah, J.M., Okpara, H.M., Ekanem, E.E., & Jacob Udo, J.J. (2015). Seroprevalence and predictors of hepatitis A infection in Nigerian children. 20 (120), 5501.
67. Oyefolu AOB, Ashaka OS, Anjorin AA, Salu OB, Akinyemi KO, Omilabu SA and Gbadegesin A (2016): Sero-prevalence of Hepatitis C Virus in HIV Seropositive Children in Lagos, Nigeria. African Journal of Microbiology Research; 10 (2): 62 – 65
68. Salu, O. B, Oyefolu, A. O. B, Gbadegesin, A, James A. B, Oke, B. O, Ashaka, O. S, Anjorin, A. A, Akinyemi, K. O and Omilabu S. A. (2018). Coinfection of Hepatitis B and C viruses among human immunodeficiency virus infected children in lagos, nigeria. African journal of clinical and experimental microbiology. May 2018 ISBN 1595-689x volume 19 no.2
69. Okagbue, H.I, Adamu M.O, Bishop S.A, Oguntade P.E, Odetunmibi O.A and Opanuga S.A, (2019). Hepatitis E Infection in Nigeria: A systematic Review. *Open Access Macedonian Journal of Medical Sciences* 7(10): 1719- 1722.
70. Fowotade A, Akande O, Gbaja A.T, Ogunleye V.O, Ajayi A and Kehinde A.O (2018). Seroprevalence of hepatitis E among restaurant food handlers in Ibadan. *Tanzania journal of health* 20(3):1-7.
71. Buti, M, Domínguez, A.P., Plands, P., Jardí, R., Schaper, M., Espuñes, J., Cardeñosa, N., Rodríguez-Frías, F., Esteban, R., Plasència, A. & Salleras, L. (2006). Community-based seroepidemiological survey of hepatitis E virus infection in Catalonia, Spain. *Clinical Vaccine Immunology*13:1328-1332.

Table 1: Primer Sequences for HAV, HBV, HCV and HEV Reverse Transcription (RT-PCR) and PCR amplification.

Virus	Primer	Sequences	Amplicon size (bp)
**Hepatitis A Virus**	HAVC-R, 5'<br>HAVC-L, 5'	CTCCAGAATCATCTCCAAC-3'<br>CAGCACATCAGAAAGGTGAG-3'	**192bp**
**Hepatitis B Virus**	P2f<br>979<br>Mc2r_(rv)	5'-CCT GCT GGT GGC TCC AGT TC (20)-3'<br>5'-CAA AAG ACC CAC AAT TCT TTG ACA TAC TTT CCA AT (35)-3'<br>5'-TGG AAG TTG GGG ATC ATT GC (20)-3'	**1000+bp**
**Hepatitis C Virus**	HCV_A1<br>HCV_S1<br>HCV_A2<br>HCV_S2	5'-GAT GCA CGG TCT ACG AGA CCT C (22)-3'<br>5'-AAC TAC TGT CTT CAC CCA GAA (21)-3'<br>5'-GCG ACC CAA CAC TAC TCG GCT (21)-3'<br>5'-ATG GCG TTA GTA TGA GTG (18)-3'	**200bp**
**Hepatitis E Virus**	ORF1F1 strand<br>ORF1R1 strand<br>ORF1Fnstd<br>ORF1Rnstd	5'-CCA YCA GTT YAT HAA GGC TCC- 3' (21mer)<br>5'-TAC CAV CGC TGR ACR TC-3' (17mer)<br>5'-CTC CTG TTG GCR TYA CWA CTE C- 3' (19mer)<br>5'- GGR TGR TTC CAI ARV ACY TC- 3 (20mer)	**170bp**

Click here to access/download;Figure;Table 2.jpg

Table 2: Socio-Demographics Characteristics of Study Participants (n=350)

PARAMETER	LEVEL	FREQUENCY (%)
GENDER	MALE	212 (60.6)
	FEMALE	138 (39.4)
AGE	<1	4 (1.1)
	1-10	65 (18.6)
	11-20	71 (20.3)
	21-30	98 (28.0)
	31-40	66 (18.9)
	41-50	31 (8.9)
	51-60	9 (2.6)
	61-70	6 (1.7)
GEO-POLITICAL ZONE	NORTH-CENTRAL	26 (7.4)
	NORTH-EAST	120 (34.3)
	NORTH-WEST	54 (15.4)
	SOUTH-EAST	1 (0.3)
	SOUTH-SOUTH	26 (7.4)
	SOUTH-WEST	123 (35.1)
SYMPTOMS	FEVER	306 (87.4)
	FEVER/BLEEDING	44 (12.6)
LIVE STATUS	DEAD	28 (8.0)
	ALIVE	322 (92.0)

Table 3: Distribution of Hepatitis A, B, C and E Positivity among the participants and their Geographic Location (n=350).

VARIABLES	POSITIVE (%)
**Hepatitis Status**	
HAV	0 (0.0)
HBV	37 (10.6)
HCV	18 (5.1)
HEV	71 (20.3)
**Total**	126 (36)
**Hepatitis Coinfection**	
HBV/HCV	3 (20.0)
HBV/HEV	2 (13.3)
HCV/HEV	8 (53.3)
HBV/HCV/HEV	2 (13.3)
**Total**	15 (11.0)
**GENDER**	
MALE	79 (62.7)
FEMALE	47 (37.3)
**AGE GROUP**	
<1	0 (0.0)
1-10	15 (4.3)
11-20	31 (8.9)
21-30	36 (10.3)
31-40	21 (6.0)
41-50	16 (4.6)
51-60	5 (1.4)
61-70	2 (0.6)
**LOCATION**	
NORTH-EAST	67 (19.1)
NORTH-WEST	23 (6.6)
NORTH-CENTRAL	7 (26.9)
SOUTH-WEST	22 (6.2)
SOUTH-SOUTH	7 (2.0)
SOUTH-EAST	0 (0.0)

Table 4: Hepatitis Co-infection Distribution in Geographic Regions and Gender

REGIONS	No. of Samples (%)		Total (%)	No. of +ve Samples (%)		Total
	**Male**	**Female**		**Male**	**Female**	
**N/EAST**	63 (29.7)	57 (41.3)	120 (34.3)	3 (37.5)	3 (42.9)	**6 (40%)**
**N/WEST**	38 (17.9)	16 (11.6)	54 (15.4)	2 (25)	1 (14.3)	**3 (20%)**
**N/CENTRAL**	12 (5.7)	14 (10.1)	26 (7.4)	-	-	-
**S/WEST**	85(40.1)	38(27.5)	123 (35.1)	2(25)	1(14.3)	**3 (20%)**
**S/SOUTH**	14(6.6)	12(8.7)	26 (7.4)	1(12.5)	2(28.6)	**3 (20%)**
**S/EAST**	-	1(0.7)	1 (0.3)	-	-	-
**TOTAL**	**212 (60.6)**	**138 (39.4)**	**350 (100)**	**8 (53.3)**	**7 (46.7)**	**15 (100)**
**P Value**				**0.242**	**0.285**	

Click here to access/download;Figure;Figure 1 HBV.jpg

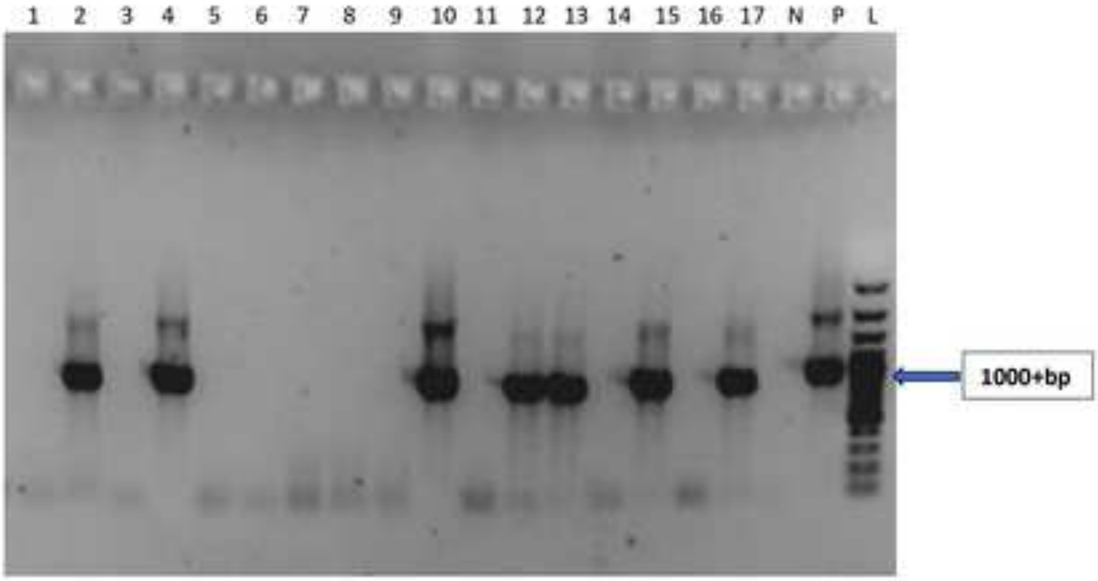

Figure 1: Agarose gel image showing the PCR amplicon bands for Hepatitis B Virus DNA. Note: Samples on lanes 2, 4, 10, 12, 13, 15 and 17 were positive with an amplicon size of 1000+bp as compared with the positive control. N – Negative Control; P – Positive Control and L – DNA ladder (100bp).

Figure

Click here to access/download;Figure;Figure 2 HCV.jpg

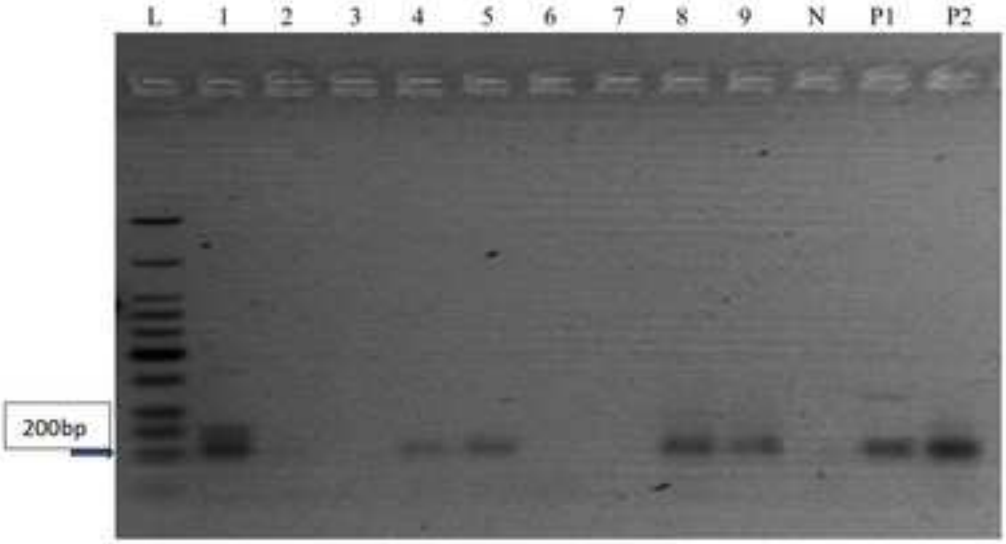

Figure 2: Agarose gel image showing the PCR amplicon bands for Hepatitis C Virus RNA. Note: Samples on lanes 1, 4, 5, 8 and 9 were positive with an amplicon size of 200bp as compared with the positive control. N – Negative Control; P1 & P2 – Positive Control and L – DNA ladder (100bp).

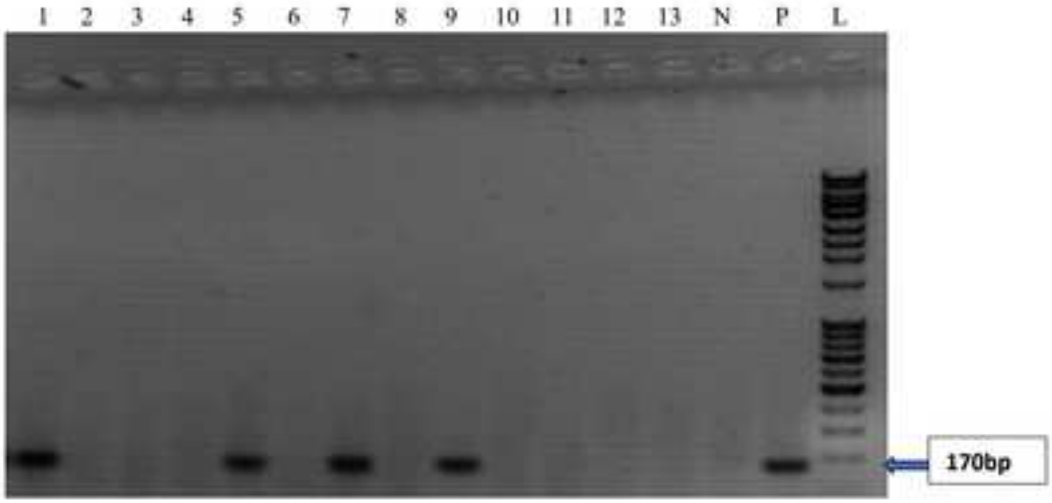

Figure 3: Agarose gel image showing the PCR amplicon bands for Hepatitis E Virus RNA. Note: Samples on lanes 1,5,7 and 9 were positive with an amplicon size of 170bp as compared with the positive control. N – Negative Control; P – Positive Control and L – DNA ladder (100bp).

 Click here to access/download;Figure;Figure 4 HAV.jpg

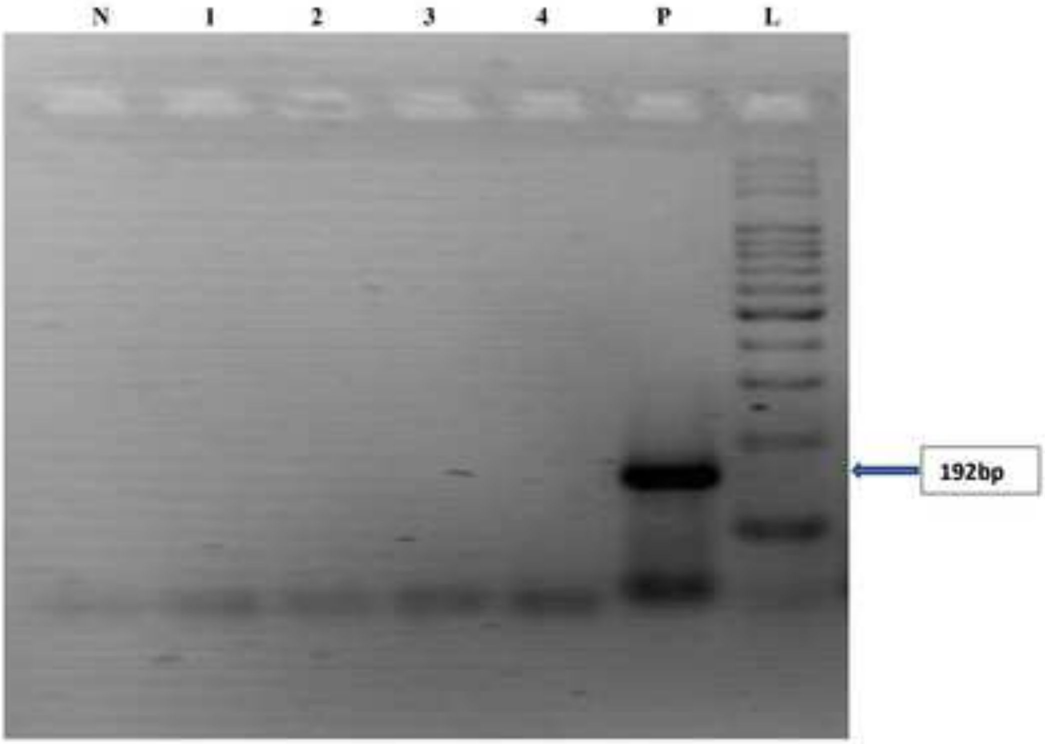

Figure 4: Agarose gel image showing the PCR amplicon bands for Hepatitis A Virus RNA. Note: Samples on lanes 1, 2, 3 and 4 were negative as compared with the positive control (192bp). N – Negative Control; P – Positive Control and L – DNA ladder (100bp).

Click here to access/download
Supporting Information
Supporting Information.docx
