# Supplementary material for: Detection of hepatitis viruses in suspected cases of Viral Haemorrhagic Fevers in Nigeria
Source: PLoS One. 2024 Jun 21;19(6):e0305521. doi: 10.1371/journal.pone.0305521 (PMC11192311; doi:10.1371/journal.pone.0305521)
Supplement: Attachment — Submitted filename: Response to Reviewers.docx [file pone.0305521.s003.docx]

**Authors Responses to Comments**

**PONE-D-23-34199**

Detection of Hepatitis Viruses in Suspected Cases of Viral Haemorrhagic Fevers in Nigeria.

**RESPONSE REVIEWRS’ COMMENTS**

| **Journal Additional Requirements** | **Author Response** |
| --- | --- |
| 1. Please ensure that your manuscript meets PLOS ONE's style requirements, including those for file naming. The PLOS ONE style templates can be found at https://journals.plos.org/plosone/s/file?id=wjVg/PLOSOne_formatting_sample_main_body.pdf and https://journals.plos.org/plosone/s/file?id=ba62/PLOSOne_formatting_sample_title_authors_affiliation. | The PLOS ONE’s style requirements including those for file naming has been addressed by the authors to the best of their abilities. |
| 2. Please include captions for your Supporting Information files at the end of your manuscript, and update any in-text citations to match accordingly. Please see our Supporting Information guidelines for more information: http://journals.plos.org/plosone/s/supporting-information | Captions for the Supporting Information files has been included at the end of the manuscript.  In-text citations has been updated to match accordingly. |
| **Reviewers Comments** | **Author Response** |
| Reviewer 1 |  |
| The identification of the different causes of febrile illness is the topic of many publications in Africa and is not at all a trivial issue (see for example M.J. Maze et alii, The epidemiology of febrile illness in sub-Saharan Africa: implications for diagnosis and management. Clinical Microbiology and Infection 24, 2018, 808-814). What is the proportion of samples routinely considered as negative for LASV, YFV or DENV in the laboratory of the authors? | The proportion of samples routinely confirmed as negative for LASV, YFV or DENV at our facility: The Centre for Human and Zoonotic Virology over the last 2 decades is approximately 93.5%. This has been included in the last paragraph of background. |
| The choice of the serological tests is puzzling. The authors targeted acute hepatitis A by using IgM anti-HAV kit, but they looked in the meantime for markers used to detect chronic diseases for HCV and HBV or for past disease for HEV. They are thus mixing interpretations for acute and chronic hepatitis. It is probable that all anti-HCV or HBsAg positive patients were chronically infected for a while and that the fever that motivated the haemorrhagic fever diagnosis was not due to HBV or HCV. | Both IgM antibodies were assayed for HAV and HEV and not IgG for HEV as specified in the query. While the total antibodies (IgM, IgG and IgA) were assayed for HCV.  Truly, the fever in these suspected cases of VHF were could not be ascertained to be directly due to these hepatitis viruses detected.  This statement has been rephrased in the first paragraph of the discussion to highlight that further evaluation is required to ascertain the actual contributions of these viruses as co-morbidities and co-mortalities in VHF cases. |
| The 5% and 10% of smaples positive for HCV and HBV correspond roughly to what can be expected in the general Nigerian population while these figures should have been higher if they have been associated with the current disease. In addition, looking for anti-HEV IgG is also looking for past infection. Why not using anti-HBc IgM or anti-HEV IgM to detect an acute infection. | As stated above, both IgM antibodies were assayed for HAV and HEV and not IgG for HEV as specified.  The reason for the PCR for these viruses was to confirm the diagnosis. Results of the PCR is as highlighted in Table 5. |
| Why not looking for other HFV such as Chikungunya or West Nile Virus involved at high frequency in a former Nigerian survey (see Baba M et al. Evidence of arbovirus co-infection in suspected febrile malaria and typhoid  patients in Nigeria. J Infect Dev Ctries 2013;7:51e9) ? | The scope of this study was limited to only 4 hepatitis viruses were covered in this study.  Another study is currently ongoing to determine the various circulating agents in febrile illness and VHFs in the six geographic regions of Nigeria. |
| “The cold chain of the samples was maintained, and randomly selected plasma samples”: what was the system of selection? It can significantly impact the final outcome of the study for example if a large proportion of sample coming from the North-East region has been “selected”. | Approximately 20% of all negative samples from the various Lassa treatment facilities were randomly to minimize bias. This has been included in the methodology section. |
| Please provide a table comparing the samples positive and negative for hepatitis viruses with the outcome of statistical tests. | Table describing and comparing the demographics characteristics of the positives for HBV, HCV and HEV provided (See Table 5) |
| Please provide a table describing each type of patients: HBsAg(+), anti-HCV(+), anti-HEV(+). | Table describing and comparing the demographics characteristics of the positives for HBV, HCV and HEV provided (See Table 5). |
| There is a lengthy paragraph about the co-infections that represent only 5% of the whole series. They are only 15, and it is not sure that relevant conclusions can be draw from such a small subset. | Paragraph has been rephrased to highlight that more studies are required to establish the co-circulation of these agents. |
| Were some of the sample’s part of outbreaks or were they only sporadic cases of suspected VHF? | Samples were from both outbreaks and sporadic cases seen at the VHF treatment facilities in Nigeria. Information has been included in study design/location/population section of the materials and methods. |
| “In endemic areas, the knowledge of these hepatitis and other viruses that might be cocirculating in suspected cases of VHFs »: the provided evidence do not support the co-circulation of hepatitis and VHF in Nigeria. Regarding the co-  circulation of hepatitis viruses it is well documented by the publication of papers dedicated to co-infections. | This has been rephrased as “In endemic areas, more studies to ascertain the actual burden, co-circulation and clinical implications of hepatitis and other viruses with febrile manifestations as VHFs is advocated in Nigeria. Once established, it might be reasonable to screen for these viruses in suspected cases of VHFs since signs and symptoms are superimposed on each other which could lead to missed diagnosis” |
| Likewise, “The study also shows that there might be possible co-morbidity between viral hemorrhagic fevers and hepatitis viruses” is not a realistic statement. The study is not showing that, and it was not meant to do so. | Statement has been deleted. manuscript (See conclusion section) |
| “This finding supports the documented evidence that globally, over 350 and 150 million people are chronically infected with HBV and HCV respectively”: I do not think that the findings of the present manuscript support these figures. | Statement has been deleted. manuscript (See discussion paragraph 4). |
| We do not know anything about the percentage of positivity by PCR for any viruses. | The percentages of the positive by PCR for HBV, HCV and HEV has been included in the write as highlighted on Table 5. |
| Why analyzing HAV by PCR while it was all negative by serology? | This is just for confirmation purpose. |
| We do not know whether the patients with bleeding or those who died were infected or not with hepatitis virus. We do not know their demographical status as well. | The demographic status of those positive for HBV, HCV and HEV has been documented in Table 5. Only 1 (1.4%) death and 2 (2.8%) individuals with pregnancy are among those infected with HEV. All those infected with HBV and HCV are all alive. This has been included in the results section. |
| Discussion: The long paragraph about the North East region should be shorten and substantiated by more references. “this leads to corrupt and immoral practices »: I do not think that we can deem a subset of the Nigerian youth as corrupted and immoral. For sure, some young people are susceptible to adopt practices dangerous for their own life. | Agreed. The paragraph has been shortened. Corrupt and immoral practices statement has been removed and rephrased. |
| Abstract: Please indicate clearly that the 350 samples analyzed were negative for LASV, YFV, and DENV. “126 were positive for at least one hepatitis virus” | Corrections as indicated effected in the abstract. |
| Background: “These hepatitis agents has been documented to be associated with high disease burden, morbidity, mortality and has the potential to cause outbreaks and epidemics of about 50% of hepatitis cases worldwide”. What are the cause of the other 50% of hepatitis outbreak and epidemics? It is weird. | Statement has been rephrased: “These hepatitis agents have been documented to be associated with high disease burden, morbidity, and mortality. HBV and HCV are responsible for about 90% of these mortalities, whilst the remaining 10% of mortalities are caused by other hepatitis viruses” |
| “selected sample aliquots were extracted »: What was the volume of plasma used for extraction? | 140 µL included in the statement. |
| Results: « 212 (60.6%) males and 138 (39.4%) females with a ratio of 1:1.5” Please, precise the order of sexes to calculate the ratio M:F or F:M? | The statement has been rephrased and the precise order of sexes ratios has been corrected in other sections of the results. |
| Smoking and drinking cannot be linked directly to viral hepatitis. | Agreed. Removed from the list of practices. |
| “The findings in these age groups agrees with other studies of HEV infections in Nigeria…”: What does it mean? | The statement has been rephrased for clarity. |
| Table 1: Please provide the reference of the primers used. | References of primer sets has been included in Table 1. |
| Table 2: “geo-political”, “geographical” instead? | Corrected on Table 2. |
| Reviewer 2 |  |
| Background: The literature review should encompass an examination of different diagnostic procedures, their respective limitations, as well as the challenges faced by low- and middle-income countries (LMICs) in terms of  availability and adequacy of these diagnostic tools for identifying viral hemorrhagic fevers (VHF) and other coinfection and co-morbidities. | The different diagnostic procedure, their limitations as well as the challenges in LMICs has been included in the literature review. |
| Method.  Comments:  A comprehensive table outlining the clinical data pertaining to the signs, symptoms, and specific regions of the participants should be incorporated within the methods section. | Table 2 in the results section has been expanded to include a comprehensive demographic data of all participants |
| The population delineation by the author was insufficient. It is imperative for the authors to provide a comprehensive definition of this population, taking into account factors such as age group, pregnant and non-pregnant individuals,  with particular attention to the implications on HEV. | Comprehensive definition based on available data has been delineated on Table 2 as required. |
| Does the author think the coinfections, co-circulation, low or high prevalence could potentially be attributed to antibody dependence enhancement (ADE)? In the context of arboviruses, is it plausible that the vector (namely mosquitoes) might have been capable of carrying both viruses, or perhaps acquiring and transmitting them simultaneously? | NO. This wasn’t evaluated in this study. |
