# Supplementary material for: Detection of hepatitis viruses in suspected cases of Viral Haemorrhagic Fevers in Nigeria
Source: PLoS One. 2024 Jun 21;19(6):e0305521. doi: 10.1371/journal.pone.0305521 (PMC11192311; doi:10.1371/journal.pone.0305521)
Supplement: Attachment — Submitted filename: Response to Reiviewers.docx [file pone.0305521.s004.docx]

**Authors Responses to Comments**

**PONE-D-23-34199**

Detection of Hepatitis Viruses in Suspected Cases of Viral Haemorrhagic Fevers in Nigeria.

**RESPONSE REVIEWRS’ COMMENTS**

| **Journal Additional Requirements** | **Author Response** |
| --- | --- |
| 1. Please review your reference list to ensure that it is complete and correct. If you have cited papers that have been retracted, please include the rationale for doing so in the manuscript text or remove these references and replace them with relevant current references. Any changes to the reference list should be mentioned in the rebuttal letter that accompanies your revised manuscript. If you need to cite a retracted article, indicate the article’s retracted status in the References list and also include a citation and full reference for the retraction notice. | The reference list has been reviewed for completeness and correctness. Please note the following:   1. Reference number 16 was updated by the “Erratum” version published in March 2017. Thus, replacing the earlier version published in 2014. 2. Reference number 22 was updated and replaced by the now published version in Future Virology Journal in 2021. Thus, replacing the 2020 preprint version earlier cited. 3. Reference number 59 was removed from the manuscript as it has no relevance where cited. |
|  |  |
| **Reviewers Comments** | **Author Response** |
| Reviewer 4 |  |
| The manuscript is well research and well written. However, the statistical methods used for the analysis was not stated, and there some grammatical errors that will need to be corrected; for example: | 1. The statistical methods used for the analysis of data have been included in the methodology section (See Page 9). 2. The manuscript has been reviewed again and all grammatical errors corrected. |
| Page 6-Ethical considerations, line 4, "accessed protected" should be edited to "access-protected". | “accessed protected” edited to “access-protected” |
| Page 7-Data/specimen retrieval and storage, line 3, "randomly by......" should be edited to "randomly selected by.....". | “randomly by…..” edited to “randomly selected by…..” |
